# Feeding Habits and Trophic Level of the Panama Grunt *Pomadasys panamensis*, an Important Bycatch Species from the Shrimp Trawl Fishery in the Gulf of California

**DOI:** 10.1155/2014/864241

**Published:** 2014-10-14

**Authors:** José A. Rodríguez-Preciado, Felipe Amezcua, Brian Bellgraph, Juan Madrid-Vera

**Affiliations:** ^1^Posgrado en Ciencias del Mar y Limnología, Universidad Nacional Autónoma de México, Circuito Exterior s/n Ciudad Universitaria, 04510 México, DF, Mexico; ^2^Instituto de Ciencias del Mar y Limnología, Universidad Nacional Autónoma de México, Avenida Joel Montes Camarena s/n, 82040 Mazatlán, SIN, Mexico; ^3^Pacific Northwest National Laboratory, P.O. Box 999, Richland, WA 99352, USA; ^4^Instituto Nacional de Pesca, Centro Regional de Investigación Pesquera Mazatlán, Avenida Sábalo-Cerritos s/n, 82100 Mazatlán, SIN, Mexico

## Abstract

The Panama grunt is an abundant and commercially important species in the southeastern Gulf of California, but the research undertaken on this species is scarce despite its ecological and economic importance. We studied the feeding habits of Panama grunt through stomach content analyses as a first step towards understanding the biology of this species in the study area. Our results indicate that the Panama grunt is a benthic predator throughout its life cycle and feeds mainly on infaunal crustaceans. Diet differences among grunt were not found according to size, diet, or season. Shannon diversity index results indicate that Panama grunt has a limited trophic niche breadth with a diet dominated by a limited number of taxa as crustaceans. The estimated trophic level of this species is 3.59. Overall, the Panama grunt is a carnivorous fish occupying the intermediate levels of the trophic pyramid.

## 1. Introduction

An adequate conservation program requires basic ecological parameters of the exploited resources to enable educated decision-making for the future management of a fishery. Feeding studies can be used in fisheries research to integrate diet results with appropriate fisheries models, such as multispecies virtual population analysis, and can be scaled to estimate total biomass of predators and prey, information which provides estimates of the total biomass consumed by predators [1]. Also, the feeding ecology of exploited fish species is essential for understanding issues such as resource-partitioning and within- and between-species competition, prey selection, predator-prey size relationships, ontogenetic dietary shifts, habitat selection, and invasions (e.g., [2–8]). Further, feeding-habit studies are necessary for the ecosystem-based management of aquatic resources, through the estimation of trophic levels [9, 10]. The problem is that, in general, little is known about trophic interactions between exploited fish species and other organisms in the ecosystem or of the factors determining the strength of predator-prey and competitive interactions [11]. Therefore, the compilation of stomach content data is an important step towards the development of ecosystem models and various modeling tools.

In this work, the feeding habits of the Panama grunt (*Pomadasys panamensis*) were studied. This is a widely distributed species in the Tropical Eastern Pacific (TEP) that inhabits sandy and rocky seabed in estuarine areas along the continental shelf at depths less than 50 m. In the southeastern Gulf of California of Mexico, this species is abundant and is subject to commercial exploitation throughout the year by artisanal fishermen using trawl and gill nets [12, 13], and from September to March by the shrimp-trawl fishery, which discards grunt as bycatch [14]. Panama grunt is one of the main components of the shrimp fishery bycatch, accounting for 6.3% of the biomass and 3.7% of the abundance of the demersal fish species caught in the southeastern Gulf of California. This species as bycatch is equivalent to approximately 30 tones per trip, with an economic value of $25,000.00 USD; value that is typically discarded and lost to the artisanal fishery. Despite the economic loss to this and other estuarine systems in the TEP, the bycatch of Panama grunt is currently unregulated, and little is known about its ecology; in particular, studies on its diet and feeding habits are scarce.

The objectives of the present study were to present detailed information on the diet composition, diversity, and trophic level of the Panama grunt. Moreover, this study provides basic data for the development of multispecies assessment models with the ultimate goal of developing an ecosystem-based management plan (EBMP) that can be applied to the demersal fish community of the southeastern Gulf of California and the entire TEP. Ideally, this EBMP would restore and protect the health, function, and resilience of entire ecosystem. Because such plan needs to have scientifically defined boundaries in order to focus on a specific zone, as well as to account for the ways in which the different organisms in that zone interact, the studies on feeding habits are basic towards the elaboration of such plan as they allow us to know these interactions, as well as to defining boundaries where the feeding and therefore the condition of the fishes are optimal.

## 2. Material and Methods

**Figure 1:**
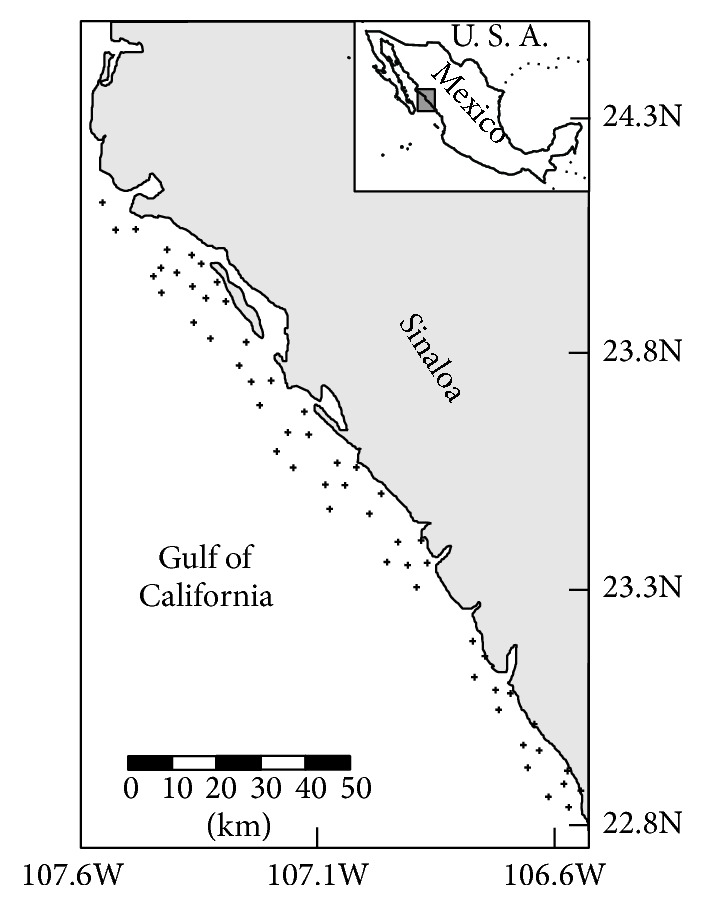
Map of the study area in the southeast Gulf of California, Mexico, and sampling areas (+) where Panama grunt was sampled.

Monthly demersal fishing surveys aboard commercial vessels from 2002 to 2007 were carried out by the National Fisheries Institute of Mexico (INAPESCA) along the coast of Sinaloa (southeastern Gulf of California, Figure 1) to capture specimens for diet analyses. A stratified survey design (by depth and area) with fixed sampling positions was used. In each survey, 59 stations were sampled over a two-week period with two commercial trawls fitted with a 30 mm cod-end mesh liner and an average door spread of 34.9 m. Trawls were towed at 2.3 knots for 60 minutes during daylight hours. After each tow collection, individuals of* P. panamensis* were immediately frozen on board at a temperature not higher than −25°C to minimize microbial and enzymatic activity [15]. Because the samples come from the commercial shrimp trawlers, solutions such as formaldehyde are not allowed on board, as these could contaminate the shrimp and fish used for commercial purposes.

In the laboratory, total length (TL; cm) and wet weight (g) were recorded for each specimen. Each fish was then dissected and sexed, and the stomachs were removed and preserved in 4% formalin. Stomach contents were identified under a stereoscopic microscope. Where possible, prey items were identified to species; however, they were typically identified to family or the lowest taxonomic level possible due to having been partially digested. Next, diet items were counted and weighed to the nearest milligram after removal of surface water. For analyses, prey items were divided into groups similar to those used by Langton and Watling [16]. These groups took account of the taxonomy of the different prey items as well as their life history traits (e.g., mobility, size, and morphological relationships); the way that the prey might be perceived by Panama grunt was also considered.

Seasonal feeding intensity was assessed using the vacuity index (VI) and the repletion index (RI). The VI is the proportion of empty stomachs via the formula

(1) $$\begin{array}{c} \mathrm{VI}=\left(\frac{N_{\text{es}}}{T_{\text{s}}}\right)\times 100, \end{array}$$

where *N*_es_ is the number of empty stomachs and *T*_s_ is the total number of stomachs. A *Z* test was then used to determine if significant differences occurred seasonally in VI. The formula includes an estimate of the complementary proportion of each sampling station:

(2) $$\begin{array}{c} Z=\left(\frac{{\overset{-}{X}}_{1}-{\overset{-}{X}}_{2}}{\hat{p}\hat{q}\sqrt{S_{1}^{2}/n_{1}+S_{2}^{2}/n_{2}}}\right), \end{array}$$

where $\overset{-}{X}$ is mean, *S* ^2^ is variance, *n* is the total number of stomachs analyzed, and ($\hat{p}$, $\hat{q}$) is the estimation of the mean proportion that exists between empty stomachs and the total stomachs analyzed. The critical value of the *Z* test was 1.96. The stomachs were obtained from different hours throughout the day, and no intent was made to account for diel effects. Assuming that the samples were randomly obtained, the mean VI and its standard deviation should include the error associated with feeding at different rates through the day.

A randomized cumulative species curve was constructed to determine if the number of stomachs analyzed was sufficient to describe the total number of prey species expected in our samples [17]. The order in which samples were analyzed was randomized 1,000 times. For each new cumulative species sample, the negative exponential model proposed for species-accumulation of rare taxa [18] was adjusted by minimizing the negative-logarithmic likelihood via the equation

(3) $$\begin{array}{c} S_{t}=\beta_{0}\left(1-e^{-\beta_{1}t_{i}}\right), \end{array}$$

where *S*_*t*_ is the prey species richness of stomach *t*_*i*_, *β*_0_ is the asymptotic value of prey species richness (*S*_max⁡_) as *t* → +*∞*, and *β*_1_ is the rate at which the maximum value is attained. Parameters *β*_0_ and *β*_1_ were estimated using the Statistica 10 data analysis software system (Stat Soft Inc., 2011). For both parameters, the bias-corrected 95% confidence interval was calculated [19, 20].

To quantitatively express the importance of different prey in the diet of* P. panamensis*, the frequency of occurrence (%*O* = (number of stomachs containing prey *i*/total number of stomachs containing prey) × 100), percentage of abundance (%*N* = (number of prey *i*/total number of prey) × 100), and percentage of weight (*%W* = (weight of prey *i*/total weight of all prey) × 100) were calculated [21]. To assess prey dominance, the index of preponderance (*I*_*p*_) was used [22]. This index ranks prey in order of numerical dominance within the diet and is calculated using the formula

(4) $$\begin{array}{c} I_{p}=\frac{W_{i}\times O_{i}}{\sum \left(W_{i}\times O_{i}\right)}, \end{array}$$

where *W*_*i*_ and *O*_*i*_ are percentage weight and occurrence, respectively. For this analysis, and all those given below, only stomachs that contained food were used; empty stomachs were not used.

To evaluate niche breadth, the Shannon diversity index (*H*) was used. This index combines two quantifiable measures: species richness and species evenness. The form of the index appropriate for a finite community is

(5) $$\begin{array}{c} H=-\sum P_{i}\left({\ln P}_{i}\right)\text{,} \end{array}$$

where *P*_*i*_ is the proportion of each species in the sample. The calculation of this index was performed using the Species Diversity and Richness IV software (Pisces Conservation Ltd., 2006).

To examine dietary similarities and trophic overlap between fish length groups, seasons (based on month of sampling), and sex, a nonmetric multidimensional scaling (nMDS) analysis was applied to Bray-Curtis similarity indices between pairs of samples [23]. The data were arranged into a matrix comprising the standardized weight (g) of each prey item, and each stomach was labeled with the season (winter and summer), sex (female and male), and length group (small, 9.2–18.1 cm; medium, 18.2–27.1 cm; large, >27.1 cm). The data were fourth-root transformed to reduce the effect of very abundant prey on the analysis while retaining the quantitative nature of the data. All data were standardized to the percentage of total biomass accounted for by each species to eliminate the effect of differing sample size. Rare prey items (<4% occurrence in any sample) were removed [23]. To check for statistical evidence that the diets species composition differed among sex, length, and season, an analysis of similarity multivariate permutation test was employed using* R*-statistic values for pairwise comparisons to determine the degree of dissimilarity between groups [23]. If differences were found, a SIMPER (Similarity Percentages, PRIMER) was used in order to determine which prey categories, within each group, accounted for most of the dissimilarities within and between the levels of the tested factors when they were significantly different [23]. All analyses were performed using PRIMER 5 software.

The trophic level of the Panama grunt was estimated from diet composition data using TrophLab software (June 2000 version [24]). Both the diet composition and the trophic levels of food item(s) were considered to estimate the trophic level of* P. panamensis*. TrophLab has default trophic values for different prey items, which are based on data in FishBase [25]. TrophLab allows these values to be overwritten when better estimates are available. Trophic level of fish species *i* was then estimated from

(6) $$\begin{array}{c} \text{TROP}{\text{H}}_{i}=1+\overset{G}{\underset{j=1}{\sum }}\text{D}{\text{C}}_{ij}\times \text{TROP}{\text{H}}_{j}, \end{array}$$

where DC_*ij*_ is the proportion of prey *j* in the diet of consumer *i*, TROPH_*j*_ is the trophic level of prey *j*, and *G* is the number of groups in the diet of *i*.

A dimensionless omnivory index (OI; [26]) was calculated using the formula

(7) $$\begin{array}{c} \text{O}{\text{I}}_{i}=\overset{n}{\underset{j=1}{\sum }}{\left[\text{T}{\text{L}}_{j}-\left(\text{T}{\text{L}}_{i}-1\right)\right]}^{2}\cdot \text{D}{\text{C}}_{ij}, \end{array}$$

where TL_*j*_ is the trophic level of prey *j*, TL_*i*_ is the trophic level of predator *i*, and DC_*ij*_ is the proportion that prey *j* constitutes of the diet of predator *i*. When the value of OI is zero, the consumer in question is specialized (i.e., it feeds on a single trophic level). A large value indicates that the consumer feeds on many trophic levels. The square root of OI is the standard error of the trophic level, and a measure of the uncertainty about its precision is due to both omnivory and sampling variability. Therefore this index is the variance of the trophic level of a consumer's prey groups [25].

## 3. Results

A total of 258 stomachs of the Panama grunt were examined; 60% (*N* = 154) were from males and 40% (*N* = 104) were from females. Fish total length ranged from 9 to 34 cm, and weight varied from 12.9 to 633.2 g. The VI (percentage of empty stomachs) changed seasonally. During spring the mean VI was 26.3%, which decreased to 13.1% in summer, increased to 71.6% in autumn, and decreased to 42.4% during winter. According to the *Z* test, the mean VI during spring was statistically different from the mean VI values during summer and autumn, but not different from the winter. Mean VI during summer and autumn was significantly lesser and greater, respectively, than the mean VI in all other seasons (Table 1).

Seventeen types of prey items were identified in stomach contents and these were divided into 10 prey groups (Table 2). Items found in the stomach contents such as green algae, red or brown trunk, mangroves, and organic matter were not taken into account for analyses as these items were considered to be accidentally ingested.

**Figure 2:**
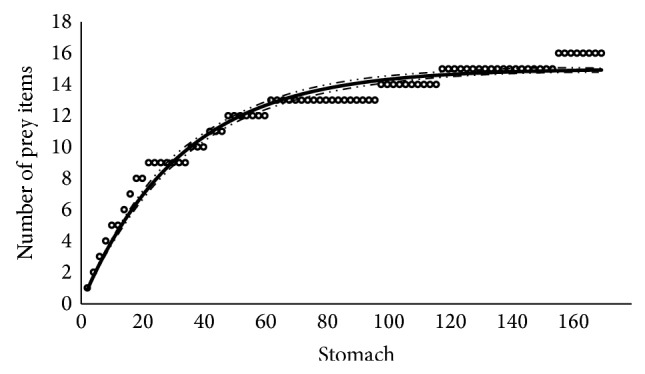
Prey item accumulation curve showing the fitted negative exponential model to diet data of Panama grunt. The form of the fitted model is *S*_*t*_ = 15(1 − *e* ^−0.062*t*_*i*_^).

The negative exponential model fitted adequately to our data and reached an asymptote at 15 prey items (Figure 2), which is less than the observed number of prey items (*N* = 17). The form of the fitted model is

(8) $$\begin{array}{c} S_{t}=15\left(1-e^{-0.062t_{i}}\right)\\ \left(t_{B\text{o}\left(166\right)}=117.3,P>0.01;t_{B1\left(166\right)}=30.1,P>0.01\right). \end{array}$$

Panama grunt had a diet dominated by benthic organisms with the main prey items consisting of shrimps (*I*_*p*_ = 0.691) as well as mysids and lobster-like crustaceans (*I*_*p*_ = 0.139 and 0.112, resp.). The remaining components of the diet were benthic invertebrates and fish (Table 2). The most abundant prey was the mysids (%*N* = 92.7), but the most frequently ingested prey, which also had the highest biomass, was penaeid shrimps (%*O* = 31.2, %*W* = 35.6). The diet breadth of the Panama grunt, according to Shannon's diversity index, was 2.03.

**Figure 3:**
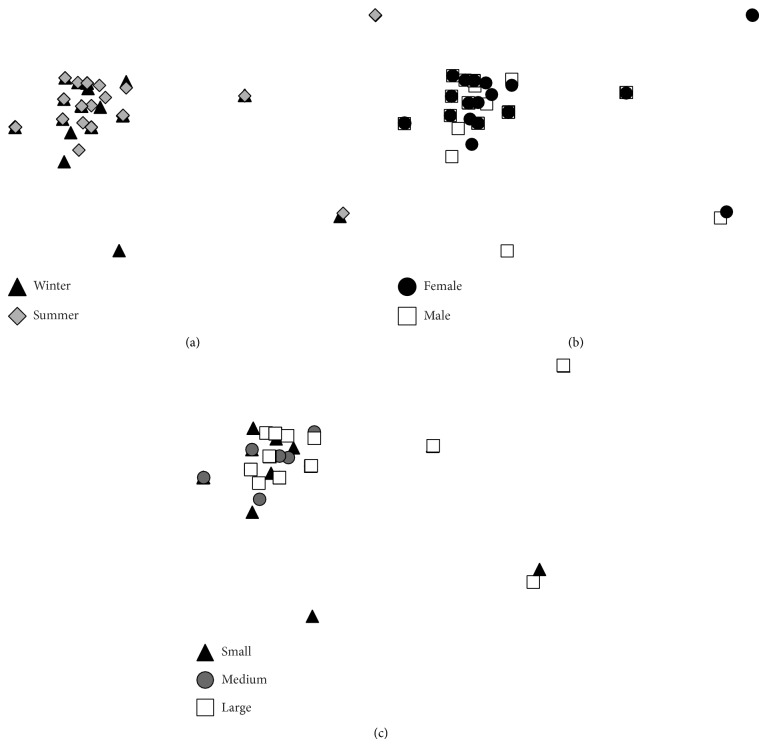
Nonmetric MDS analysis of the Panama grunt according to season (a), sex (b), and fish length (c). The stress value was 0.01.

Defined groups of length, sex, and sampling month were not observed in the nMDS plot (stress = 0.01, Figure 3). The absence of groups was confirmed by ANOSIM (*R* = −0.03, *P* = 77.6% to 86.4%); therefore, no statistically significant food habit differences occurred in Panama grunt related to length, sex, or season. Because no differences were found, the SIMPER analysis was not undertaken.

The estimated TROPH value for Panama grunt was 3.59 (SE = 0.46), and the OI was 0.21. Therefore, this species can be considered a carnivore due to the OI value being closer to 0 than to 1.

## 4. Discussion

Our results suggest that Panama grunt is a benthophagic organism, which agrees with the findings of López-Peralta and Arcila [27] for the same species off the Pacific coast of Colombia where Panama grunt fed mainly on benthic crustaceans such as amphipods and stomatopods. These similar results suggest that Panama grunt feeds mainly on benthic crustaceans and is an active and primarily benthic predator in various areas of their geographic distribution due to the abundance of errant prey (shrimp, shrimp-like crustaceans, and polychaetes) found in their diet.

No clear structure or group separation was observed on nMDS, and the ANOSIM tests show that the species composition of the diet of panama grunt does not vary significantly according to the season, length, or sex. The main prey items for this species are mainly benthic crustaceans whereas other invertebrates and fish species are marginal prey.

The Panama grunt showed periods of fasting during the fall and winter as indicated by the high percentages of empty stomachs in these seasons. From a previous study it is known that this species has a partial spawning period during fall and winter, when the hepatosomatic index decreases [28]. This could indicate that the Panama grunt uses energy resources stored in the liver for the reproductive process suggesting that energy reserves were possibly moved from the liver to the gonad, making them capital spawners [29]. With this strategy, the fish accumulates energy reserves for reproduction during the feeding period and avoids periods of competition during the spawning season. Being a species with high abundance, this strategy would prevent it from having intra- and interspecific competition for resources during the spawning season.

The Shannon diversity index for the diet of Panama grunt (*H* = 2.03) indicates that the diet of this species is not very diverse. Margalef [30] indicates that the value of this index usually falls between 1.5 and 3.5 and rarely surpasses 4. The result obtained in this study is in the lower portion of this range, which indicates that this species is a feeding specialist.

Although information on the trophic levels of other demersal fish species from the southeast Gulf of California is scarce, our results indicate that the Panama grunt (TROPH value = 3.59) is a first- to second-order predator in the southeastern Gulf of California. Consumers in marine ecosystems usually have TROPH values that vary between 2.0 (for herbivorous/detrivorous organisms) and 5.0 (for piscivorous/carnivorous organisms [31]). Demersal and benthopelagic inhabitants in the southeast Gulf of California have estimated TROPH values that range from 2.5 for species such as the flathead mullet (*Mugil cephalus*) to 4.5 for the Pacific sierra (*Scomberomorus sierra*) and the Mexican barracuda (*Sphyraena ensis* [25]). Thus, a TROPH value of 3.59 can be considered an intermediate trophic level in this ecosystem.

The OI value of the Panama grunt (0.21) indicates that this species is predominantly carnivorous. These results confirm that the Panama grunt is a carnivore that feeds on benthic crustaceans with similar trophic values [24] and could be used to implement Ecosystem Based Management of Fisheries (EBMF) in the area, as the relationship between the Panama grunt and the shrimp and other commercial important invertebrates is evident. The exploitation of each species has a direct effect on the others, as the number of prey or predators could decrease as consequence of fishing, therefore having an effect on the abundance or prey availability for the different species interrelated. Although more studies are needed to understand the biology and the relationship between all the species involved in this ecosystem in order to establish a proper EBMF, an important contribution of this work is that it is necessary to understand that the management of a single species, without considering the species with which it interacts, can cause effects on the ecosystem as a whole, such as fishing down the food web, which has been proved for another fisheries worldwide [31, 32] but has not been proved in the area of our study. The reason for this is not because it is not happening here, but rather because it is difficult to study and understand the species interactions of ecosystems in tropical and subtropical areas, as the number of species involved is much higher than in temperate waters, and also the studies undertaken on these subjects in these areas are usually scarce. However, these sorts of studies are needed in order to adequately manage and preserve the exploited resources.

## 5. Conclusions

The detailed diet information presented in this study will be useful in ecological modeling as we move toward multispecies assessments and a better understanding of the ecological interactions among predators and their prey. These results, in turn, will contribute to a better understanding of the trophic flows associated with demersal fish in the Gulf of California. Nevertheless, to achieve an understanding of the larger ecosystem, it will be necessary to continue with trophic studies for other species inhabiting the area. Monitoring activity of fishery landings, fishing efforts, and variations in biotic and abiotic factors in the area over the long-term will also assist in realizing an ecosystem approach to fisheries.

## Figures and Tables

**Table 1:** Pairwise comparisons between seasonal values of the mean vacuity index (VI) using *z*.

Season	*z*	*P* value
Spring-summer	4.07	<0.05
Spring-autumn	4.1	<0.05
Spring-winter	0.55	>0.05
Summer-autumn	6.8	<0.05
Summer-winter	3.94	<0.05
Autumn-winter	3.49	<0.05

**Table 2:** Indices describing the importance of functional prey groups of *Pomadasys panamensis* as described by the index of preponderance (*I*_*p*_), percentage abundance (%*N*), frequency of occurrence (%*O*), and percentage weight (%*W*).

Group	Prey taxa	*I*_*p*_	%*N*	%*O*	%*W*
Shrimp	Penaeidae, Caridea	0.691	2.4	31.3	38.7
Mysids	Mysidae	0.139	92.7	8.6	26
Lobster = like	Stomatopoda, Achelata	0.112	1.1	17.2	13.1
Errant polychaete	Phyllodocida	0.020	1.1	10.7	3.2
Octopi	Cephalopoda	0.012	0.2	5.4	3.7
Crabs	Paguroidea, Portunidae	0.009	0.3	6.2	4.7
Fish	Anguillidae, Engraulidae, Clupeidae, and Sciaenidae	0.008	1.1	8.7	5.5
Brittle stars	Asteroidea, Ophiuroidea	0.005	0.4	4.3	2.3
Infaunal crustacean	Amphipoda	0.003	0.3	7.5	0.7
Snails	Muricidae	0.001	0.4	0.1	2.1

## References

[1] Jennings S., Kaiser M. J., Reynolds J. D. (2001). *Marine Fisheries Ecology*.

[2] Harmelin-Vivien M. L., Kaim-Malka R. A., Ledoyer M., Jacob-Abraham S. S. (1989). Food partitioning among scorpaenid fishes in Mediterranean seagrass beds. *Journal of Fish Biology*.

[3] Stergiou K. I., Fourouni H. (1991). Food habits, ontogenetic diet shift and selectivity in* Zeus faber* Linnaeus, 1758. *Journal of Fish Biology*.

[4] Hughes R. N., Godin J. G. J. (1997). Diet selection. *Behavioral Ecology of Teleost Fishes*.

[5] Labropoulou M., Machias A., Tsimenides N., Eleftheriou A. (1997). Feeding habits and ontogenetic diet shift of the striped red mullet, *Mullus surmuletus* Linnaeus, 1758. *Fisheries Research*.

[6] Labropoulou M., Machias A. (1998). Effect of habitat selection on the dietary patterns of two triglid species. *Marine Ecology Progress Series*.

[7] Pauly D., Froese R., Pauly R. D. (2000). Predator-prey ratios in fishes. *FishBase 2000: Concepts, Design and Data Sources*.

[8] Scharf F. S., Juanes F., Rountree R. A. (2000). Predator size: prey size relationships of marine fish predators: interspecific variation and effects of ontogeny and body size on trophic-niche breadth. *Marine Ecology Progress Series*.

[9] Pauly D., Christensen V., Froese R., Pauly R. D. (2000). Trophic levels of fishes. *FishBase 2000: Concepts, Design and Data Sources*.

[10] Pauly D., Sala P., Froese R., Pauly R. D. (2000). Estimating trophic levels from individual food items. *FishBase 2000: Concepts, Design and Data Sources*.

[11] Garrison L. P., Link J. S. (2000). Dietary guild structure of the fish community in the Northeast United States continental shelf ecosystem. *Marine Ecology Progress Series*.

[12] Amezcua-Linares F. *Peces demersales del Pacífico de México*.

[13] Fischer W., Krupp F., Schneider W., Sommer C., Carpenter K. E., Niem V. H. (1995). *Guía FAO para la identificación de especies para los fines de la pesca: Pacífico centro-oriental*.

[14] Madrid-Vera J., Amezcua F., Morales-Bojórquez E. (2007). An assessment approach to estimate biomass of fish communities from bycatch data in a tropical shrimp-trawl fishery. *Fisheries Research*.

[15]  SENASICA (2010). *Buenas Prácticas de Manejo a bordo para personal que trabaja en embarcaciones camaroneras [Manual informativo]*.

[16] Langton R. W., Watling L., Barnes M., Gibson R. N. The fish-benthos connection: a definition of prey groups in the Gulf of Maine.

[17] Flather C. H. (1996). Fitting species-accumulation functions and assessing regional land use impacts on avian diversity. *Journal of Biogeography*.

[18] Miller R. I., Wiegert R. G. (1989). Documenting completeness, species-area relations, and the species-abundance distribution of a regional flora. *Ecology*.

[19] Efron B., Tibshirani R. J. (1993). *An Introduction to the Bootstrap*.

[20] Haddon M. (2001). *Modeling and Quantitative Methods in Fisheries*.

[21] Hyslop E. J. (1980). Stomach contents analysis—a review of methods and their application. *Journal of Fish Biology*.

[22] Marshall S., Elliott M. (1997). A comparison of univariate and multivariate numerical and graphical techniques for determining inter- and intraspecific feeding relationships in estuarine fish. *Journal of Fish Biology*.

[23] Clarke K. R., Warwick R. M. (1994). *Change in Marine Communities: an Approach to Statistical Analysis and Interpretation*.

[24] Pauly D., Froese R., Sala P. S., Palomares M. L., Christensen V., Rius J. (2000). *TrophLab Manual*.

[25] Froese R., Pauly R. D. http://www.fishbase.org/.

[26] Christensen V., Pauly D. (1992). ECOPATH II—a software for balancing steady-state ecosystem models and calculating network characteristics. *Ecological Modelling*.

[27] López-Peralta R. H., Arcila C. A. T. (2002). Diet composition of fish species from the southern continental shelf of Colombia. *Naga*.

[28] Lara-Mendoza R. E. (2011). *Edad, crecimiento y reproducción de Pomadasys panamensis y Haemulopsis leuciscus (Pisces : Haemulidae) del sureste del Golfo de California durante 2002–2009 [MSc Dissertation]*.

[29] Rijnsdorp A. D. (1994). Population-regulating processes during the adult phase in flatfish. *Netherlands Journal of Sea Research*.

[30] Margalef R. (1972). Homage to Evelyn Hutchinson, or why there is an upper limit to diversity. *Transactions of the Connecticut Academy of Arts and Sciences*.

[31] Cortés E. (1999). Standardized diet compositions and trophic levels of sharks. *ICES Journal of Marine Science*.

[32] Pauly D., Christensen V., Dalsgaard J., Froese R., Torres F. (1998). Fishing down marine food webs. *Science*.

